# Chemotherapy in the treatment of different histological types of appendiceal cancers: a SEER based study

**DOI:** 10.1186/s12885-021-08502-3

**Published:** 2021-07-06

**Authors:** Gang Wang, Qiken Li, Weiping Chen

**Affiliations:** Department of Colorectal Surgery, Cancer Hospital of the University of Chinese Academy of Sciences, Zhejiang Cancer Hospital, Hangzhou, 310022 China

**Keywords:** Appendiceal cancer, Chemotherapy, Histology, Survival, Adenocarcinoma, Neuroendocrine

## Abstract

**Background:**

Due to its rarity and high heterogeneity, neither established guidelines nor prospective data are currently available for using chemotherapy in the treatment of appendiceal cancer. This study was to determine the use of chemotherapy and its potential associations with survival in patients with different histological types of the cancer.

**Methods:**

Patients with histologically different appendiceal cancers diagnosed during 1998–2016 were selected from the Surveillance, Epidemiology, and End Results (SEER) database. The role and effect of chemotherapy were examined in the treatment of the disease. The Kaplan-Meier method was applied to construct survival curves and significance was examined by Log-rank test. Cox proportional hazard models were used to analyze the impact of chemotherapy and other variables on survival in these patients.

**Results:**

A total of 8733 appendiceal cancer patients were identified from the database. Chemotherapy was administrated at highly variable rates in different histological types of appendiceal cancer. As high as 64.0% signet ring cell carcinoma (SRCC), 46.4% of mucinous adenocarcinomas (MAC), 40.6% of non-mucinous adenocarcinoma (NMAC) and 43.9% of mixed neuroendocrine non-neuroendocrine neoplasms (MiNENs) were treated with chemotherapy, whereas only 14.7% of goblet cell carcinoma (GCC), 5% neuroendocrine tumors (NETs) and 1.6% carcinomas (NEC) received chemotherapy. In all patients combined, chemotherapy significantly improved overall survival during the entire study period and cancer-specific survival was improved during in cases from 2012–2016. Further multivariate analysis showed that both cancer-specific and overall survival was significantly improved with chemotherapy  in patients with MAC, NMAC and SRCC, but not for patients with GCC, MiNENs, NETs and NECs. Number (> 12) of lymph node sampled was associated with survival of patients with most histological types of cancer under study. Other prognostic factors related to individual histological types were identified.

**Conclusions:**

Chemotherapy is administrated at highly variable rates in different histological types of appendiceal cancer. Efficacy of chemotherapy in the treatment of these cancers has been improved in recent years and is significantly associated with better survival for patients with NMAC, MAC, and SRCC. Adequate lymph node sampling may result in a survival benefit for most of these patients.

**Supplementary Information:**

The online version contains supplementary material available at 10.1186/s12885-021-08502-3.

## Introduction

Appendiceal cancer is a rare and highly heterogeneous malignancy and its incidence is on the rise [1]. This cancer includes a wide spectrum of histological types including: mucinous adenocarcinoma (MAC), non-mucinous adenocarcinoma (NMAC), signet-ring cell adenocarcinomas (SRCC), mixed neuroendocrine non-neuroendocrine neoplasms (MiNENs)**,** goblet cell carcinoid (GCC) neuroendocrine tumors (NETs)**,** neuroendocrine carcinomas (NECs)**,** and others [2]. These histological types display dramatically different biological phenotypes and indicate different prognoses [2, 3].

Though surgery is the first option in the treatment of appendiceal cancer, chemotherapy has been applied using a protocol similar to that used to treat colorectal cancer [3, 4]. The rarity and high heterogeneity make it difficult to examine the effect of chemotherapy in treatment of appendiceal cancer in systematic studies [5, 6]. Most previous studies had a limited sample size from a single institution, and only included certain histological types [7–10]. The effect of chemotherapy in some histological types is not well understood. In addition, it is unknown whether chemotherapy has had an improved effect in recent years.

Using the Surveillance, Epidemiology, and End Results (SEER) database, this study sought to determine the status of chemotherapy as a treatment of the different histological types of appendiceal cancer. Based on cancer-specific and overall survival outcomes, we will further determine which histological types were responsive to chemotherapy. As there are currently no standard guidelines for chemotherapy in the treatment of appendiceal cancer, the findings of this study may aid in improving the management and survival of appendiceal cancer patients.

## Patients and methods

Appendiceal cancer patients diagnosed between1998 and 2016 were selected from the Surveillance Epidemiology and End Results (SEER) database using the SEERStat software 8.3.8 [11]. Histology codes were obtained from the Third Edition of the International Classification of Diseases for Oncology (ICD-O-3). Patients with the following histological types were included: non-mucinous adenocarcinoma (NMAC) 8140, 8144, 8211, 8255, 8262, 8310, 8440 and 8460; mucinous adenocarcinoma (MAC) 8470, 8471, 8480 and 8481; goblet cell carcinoid (GCC) 8243 and 8245; signet ring cell carcinoma (SRCC) 8490; mixed neuroendocrine non-neuroendocrine neoplasms (MiNENs) 8244; neuroendocrine tumors (NETs) 8240 and 8241; neuroendocrine carcinomas (NECs) 8013 and 8246 [2]. Patients were excluded if their age at diagnosis was less than 18 years, or if their survival time or T, N, M stage information was unknown, or if they had had a tumor at Tis or T0 stage.

The following clinicopathological variables were extracted from the database: age at diagnosis, year of diagnosis, gender, race, region, status of serum CEA, tumor size, histology, tumor grade, tumor deposit, T, N and M stages, number of lymph node harvested, surgery, chemotherapy, survival time, cancer-specific death, and overall death. Race was grouped into four categories: white, black, other and unknown. Tumor size was categorized into three groups: ≤ 5 cm, > 5 cm and unknown. Number of lymph nodes harvested were divided into three groups: ≤ 12, > 12 and unknown. The extent of surgery was categorized into three groups: less than hemicolectomy, hemicolectomy or more, and unknown.

The Human Subjects Committee of Institutional Review Board in our hospital exempted this study from review since preexisting data with no personal identifiers was used.

### Statistical analysis

Continuous data were presented as mean ± standard deviation (SD), or median (range). Differences were analyzed using T test or One-Way ANOVA after a square root transformation, if necessary. Categorical data were analyzed using the Chi-square test. The survival curves were constructed using the Kaplan-Meier method and Log-rank test was applied to interrogate significant differences. Univariate and multivariate Cox proportional hazard models were used to compare the impact of chemotherapy and other variables on both cancer-specific and overall survival in appendiceal cancer patients. A backward stepwise selection was used to select variables to build multivariate models, in which chemotherapy was always included. Briefly, all variables were first included in a model. During the backward selection, a variable with the highest *P* value was removed from each step until *P* values for each variable in the final model were less than 0.05. A two-sided *p* ≤ 0.05 was considered statistically significant. All statistical analyses were completed using SAS software V9.3 (SAS Institute, Cary, NC).

## Results

A total of 8733 appendiceal cancer patients at a median age of 57 (range 18–99) years were identified from the database. The most common histological type was MAC (32.4%), followed by NMAC (20.2%), NETs (19.1%) and GCC (12.5%), whereas SRCC, NECs and MiNENs accounted for 6.6, 4.8 and 4.5%, respectively. During the study period, 1709 (19.6%) patients died of the disease and 2733 (31.3%) died from all causes (Table 1). After stratification by demographics and by whether patients had received chemotherapy, the data revealed that chemotherapy was administered at highly variable rates among different histological types. As high as 64.0% SRCC, 46.4% MAC, 43.9% MiNENs, and 40.6% NMAC patients received chemotherapy, while only 14.7% GCC, 5% NECs and 1.6% NETs patients were treated with chemotherapy. Furthermore, a significantly higher proportions of patients that received chemotherapy died of the disease (*P* < 0.0001) or from all causes (*P* < 0.0001) (Table 1).

**Table 1:** Characteristics of appendiceal cancer patients treated with or without chemotherapy in 1998–2016

Variable	All ptients	Chemotherapy status		*P* value
	(*n* = 8733)	No (*n* = 5957)	Yes (*n* = 2776)	
Age				
Mean ± SD	56.3 ± 16.4	56.2 ± 18	56.6 ± 12.3	< 0.0001
Median (range)	57 (18–99)	57 (18–99)	57 (19–89)	
≤ 56	5075 (58.1)	2885 (48.4)	1351 (48.7)	0.4273
> 56	3658 (41.9)	3072 (51.6)	1425 (51.3)	
Gender				
Male	3947 (45.2)	3276 (68.5)	1510 (31.6)	0.0603
Female	4786 (54.8)	2681 (67.9)	1266 (32.1)	
Marital status				
Married^a^	5067 (58)	3215 (63.5)	1852 (36.6)	< 0.0001
Unmarried	3262 (37.4)	2423 (74.3)	839 (25.7)	
Unknown	404 (4.6)	319 (79)	85 (21)	
Race				
African American	854 (9.8)	576 (67.5)	278 (32.6)	0.0001
White	7299 (83.6)	4994 (68.4)	2305 (31.6)	
Other	515 (5.9)	328 (63.7)	187 (36.3)	
Unknown	65 (0.7)	59 (90.8)	6 (9.2)	
Region				
West	4185 (47.9)	2899 (69.3)	1286 (30.7)	0.0803
South	2078 (23.8)	1410 (67.9)	668 (32.2)	
Midwest	824 (9.4)	535 (64.9)	289 (35.1)	
Northwest	1646 (18.9)	1113 (67.6)	533 (32.4)	
CEA				
Negative	1126 (12.9)	513 (45.6)	613 (54.4)	< 0.0001
Positive	1097 (12.6)	430 (39.2)	667 (60.8)	
Unknown	6510 (74.5)	5014 (77)	1496 (23)	
Tumor deposit				
Negative	2222 (25.4)	1391 (62.6)	831 (37.4)	< 0.0001
Positive	438 (5)	128 (29.2)	310 (70.8)	
Unknown	6073 (69.5)	4438 (73.1)	1635 (26.9)	
Tumor size				
< 2 cm	2742 (31.4)	2467 (90)	275 (10)	< 0.0001
≥ 2 cm	3668 (42)	2086 (56.9)	1582 (43.1)	
Unknown	2323 (26.6)	1404 (60.4)	919 (39.6)	
Harvested lymph nodes				
≤ 12	5098 (58.4)	3804 (74.6)	1294 (25.4)	< 0.0001
> 12	3527 (40.4)	2093 (59.3)	1434 (40.7)	
Unknown	108 (1.2)	60 (55.6)	48 (44.4)	
Histology				
GCC	1087 (12.5)	927 (85.3)	160 (14.7)	< 0.0001
MAC	2831 (32.4)	1518 (53.6)	1313 (46.4)	
NMAC	1762 (20.2)	1046 (59.4)	716 (40.6)	
SRCC	575 (6.6)	207 (36)	368 (64)	
NECs	421 (4.8)	400 (95)	21 (5)	
NETs	1667 (19.1)	1640 (98.4)	27 (1.6)	
MiNENs	390 (4.5)	219 (56.2)	171 (43.9)	
T stage				
T1	2224 (25.5)	2122 (35.6)	102 (3.7)	< 0.0001
T2	748 (8.6)	682 (11.5)	66 (2.4)	
T3	2680 (30.7)	1904 (32)	776 (28)	
T4	3081 (35.3)	1249 (21)	1832 (66)	
N stage				
N0	6984 (80)	5293 (88.9)	1691 (60.9)	< 0.0001
N1	1096 (12.6)	454 (7.6)	642 (23.1)	
N2	653 (7.5)	210 (3.5)	443 (16)	
M stage				
M0	6604 (75.6)	5267 (79.8)	1337 (20.3)	< 0.0001
M1	2129 (24.4)	690 (32.4)	1439 (67.6)	
Grade				
Well differentiated	3015 (34.5)	2475 (82.1)	540 (17.9)	< 0.0001
Moderately differentiated	2320 (26.6)	1411 (60.8)	909 (39.2)	
Poorly or un-differentiated	1403 (16.1)	576 (41.1)	827 (59)	
Unknown	1995 (22.8)	1495 (74.9)	500 (25.1)	
Surgery				
Less than hemicolectomy	3847 (44.1)	3075 (79.9)	772 (20.1)	< 0.0001
Hemicolectomy or more	4406 (50.5)	2600 (59)	1806 (41)	
Other	480 (5.5)	282 (58.8)	198 (41.3)	
Cancer specific death				
No	7024 (80.4)	5232 (74.5)	1792 (25.5)	< 0.0001
Yes	1709 (19.6)	725 (42.4)	984 (57.6)	
Overall death				
No	6000 (68.7)	4456 (74.3)	1544 (25.7)	< 0.0001
Yes	2733 (31.3)	1501 (54.9)	1232 (45.1)	

^a^Unmarried status, including divorced, separated, widowed and unmarried. *SRCC* signet ring cell carcinoma; *MAC*, mucinous adenocarcinomas; *NMAC*, non-mucinous adenocarcinoma; *MiNENs* mixed neuroendocrine non-neuroendocrine neoplasms; *GCC* goblet cell carcinoma; *NETs* neuroendocrine tumors; *NECs* neuroendocrine carcinomas

We then compared the demographic and clinicopathological characteristics among patients with different histological types (Table 2). Patients diagnosed with NETs or NECs were significantly younger at the time of diagnosis than patients with other histological types. None of NETs and only 0.5% of NECs patients had serum CEA levels measured or tumor deposit examined. It was notable that over 90% of NECs were diagnosed at the T1 or T2 stages. In contrast, much lower percentages of patients with other histological types were at these earlier stages. Significantly higher proportions of SRCC (61.4%) and MAC (57.8%) were diagnosed at T4 stage. The data also found that both NETs and NECs had significantly smaller tumor sizes, with 65.8 and 84.0%, respectively, of tumors less than 2 cm. It is noted that 72.1% NETs patients had well differentiated cancer. Both NETs and NECs had the smallest percentage of patients died of the disease or from all causes during the study period (Table 2).

**Table 2:** Characteristics of patients with different histological types of appendiceal cancer

Variable		Histological Types						*P* value
	GCC (*n* = 1087)	MAC (*n* = 2831)	SRCC (*n* = 575)	NECs (*n* = 421)	NETs (*n* = 1667)	NMAC (*n* = 1762)	MiNENs (*n* = 390)	
Age								
Mean ± SD	56.5 ± 13.6	59.6 ± 14.2	59.8 ± 12.7	45.7 ± 17.3	44.9 ± 17.8	62.8 ± 14.8	58.4 ± 12.	< 0.0001
Median (range)	56 (18–99)	60 (19–98)	59 (27–94)	46 (18–94)	44 (18–95)	63 (19–97)	58 (20–89)	
≤ 56	552 (50.8)	1181 (41.7)	243 (42.3)	300 (71.3)	1199 (71.9)	591 (33.5)	170 (43.6)	< 0.0001
> 56	535 (49.2)	1650 (58.3)	332 (57.7)	121 (28.7)	468 (28.1)	1171 (66.5)	220 (56.4)	
Gender								
Male	529 (48.7)	1272 (44.9)	238 (41.4)	156 (37.1)	642 (38.5)	912 (51.8)	198 (50.8)	< 0.0001
Female	558 (51.3)	1559 (55.1)	337 (58.6)	265 (63)	1025 (61.5)	850 (48.2)	192 (49.2)	
Marital status								
Married	650 (59.8)	1787 (63.1)	357 (62.1)	197 (46.8)	792 (47.5)	1041 (59.1)	243 (62.3)	< 0.0001
Unmarried^a^	386 (35.5)	946 (33.4)	194 (33.7)	182 (43.2)	769 (46.1)	655 (37.2)	130 (33.3)	
Unknown	51 (4.7)	98 (3.5)	24 (4.2)	42 (10)	106 (6.4)	66 (3.8)	17 (4.4)	
Race								
African American	103 (9.5)	256 (9)	55 (9.6)	34 (8.1)	135 (8.1)	236 (13.4)	35 (9)	< 0.0001
White	938 (86.3)	2341 (82.7)	478 (83.1)	359 (85.3)	1447 (86.8)	1395 (79.2)	341 (87.4)	
Other	40 (3.7)	223 (7.9)	41 (7.1)	17 (4)	60 (3.6)	122 (6.9)	12 (3.1)	
Unknown	6 (0.6)	11 (0.4)	1 (0.2)	11 (2.6)	25 (1.5)	9 (0.5)	2 (0.5)	
Region								
West	467 (43)	1506 (53.2)	275 (47.8)	171 (40.6)	782 (46.9)	820 (46.5)	164 (42.1)	< 0.0001
South	271 (24.9)	585 (20.7)	132 (23)	110 (26.1)	417 (25)	467 (26.5)	96 (24.6)	
Midwest	120 (11)	252 (8.9)	45 (7.8)	52 (12.4)	140 (8.4)	153 (8.7)	62 (15.9)	
Northwest	229 (21.1)	488 (17.2)	123 (21.4)	88 (20.9)	328 (19.7)	322 (18.3)	68 (17.4)	
CEA								
Negative	121 (11.1)	430 (15.2)	145 (25.2)	2 (0.5)	0 (0)	348 (19.8)	80 (20.5)	< 0.0001
Positive	40 (3.7)	671 (23.7)	100 (17.4)	2 (0.5)	0 (0)	254 (14.4)	30 (7.7)	
Unknown	926 (85.2)	1730 (61.1)	330 (57.4)	417 (99.1)	1667 (100)	1160 (65.8)	280 (71.8)	
Deposit								
Negative	470 (43.2)	866 (30.6)	158 (27.5)	2 (0.5)	0 (0)	564 (32)	162 (41.5)	< 0.0001
Positive	21 (1.9)	176 (6.2)	82 (14.3)	2 (0.5)	0 (0)	123 (7)	34 (8.7)	
Unknown	596 (54.8)	1789 (63.2)	335 (58.3)	417 (99.1)	1667 (100)	1075 (61)	194 (49.7)	
Tumor size								
< 2 cm	383 (35.2)	260 (9.2)	44 (7.7)	277 (65.8)	1400 (84)	312 (17.7)	66 (16.9)	< 0.0001
≥ 2 cm	392 (36.1)	1456 (51.4)	334 (58.1)	119 (28.3)	240 (14.4)	927 (52.6)	200 (51.3)	
Unknown	312 (28.7)	1115 (39.4)	197 (34.3)	25 (5.9)	27 (1.6)	523 (29.7)	124 (31.8)	
Harvested lymph nodes								
≤ 12	624 (57.4)	1589 (56.1)	282 (49)	284 (67.5)	1288 (77.3)	880 (49.9)	151 (38.7)	< 0.0001
> 12	457 (42)	1199 (42.4)	286 (49.7)	130 (30.9)	364 (21.8)	855 (48.5)	236 (60.5)	
Unknown	6 (0.6)	43 (1.5)	7 (1.2)	7 (1.7)	15 (0.9)	27 (1.5)	3 (0.8)	
T stage								
T1	108 (9.9)	233 (8.2)	20 (3.5)	293 (69.6)	1416 (84.9)	143 (8.1)	11 (2.8)	< 0.0001
T2	137 (12.6)	180 (6.4)	14 (2.4)	48 (11.4)	152 (9.1)	204 (11.6)	13 (3.3)	
T3	649 (59.7)	783 (27.7)	188 (32.7)	48 (11.4)	80 (4.8)	723 (41)	209 (53.6)	
T4	193 (17.8)	1635 (57.8)	353 (61.4)	32 (7.6)	19 (1.1)	692 (39.3)	157 (40.3)	
N stage								
N0	950 (87.4)	2387 (84.3)	289 (50.3)	337 (80.1)	1534 (92)	1236 (70.2)	251 (64.4)	< 0.0001
N1	91 (8.4)	291 (10.3)	136 (23.7)	72 (17.1)	128 (7.7)	312 (17.7)	66 (16.9)	
N2	46 (4.2)	153 (5.4)	150 (26.1)	12 (2.9)	5 (0.3)	214 (12.2)	73 (18.7)	
M stage								
M0	1003 (92.3)	1608 (56.8)	285 (49.6)	397 (94.3)	1646 (98.7)	1377 (78.2)	288 (73.9)	< 0.0001
M1	84 (7.7)	1223 (43.2)	290 (50.4)	24 (5.7)	21 (1.3)	385 (21.9)	102 (26.2)	
Grade								
Well differentiated	165 (15.2)	1054 (37.2)	11 (1.9)	304 (72.2)	1201 (72.1)	244 (13.9)	36 (9.2)	< 0.0001
Moderately differentiated	142 (13.1)	973 (34.4)	35 (6.1)	50 (11.9)	136 (8.2)	928 (52.7)	56 (14.4)	
Poorly or un-differentiated	88 (8.1)	296 (10.5)	381 (66.3)	31 (7.4)	9 (0.5)	441 (25)	157 (40.3)	
Unknown	692 (63.7)	508 (17.9)	148 (25.7)	36 (8.6)	321 (19.3)	149 (8.5)	141 (36.2)	
Surgery								
Less than hemicolectomy	489 (45)	983 (34.7)	167 (29)	246 (58.4)	1216 (73)	619 (35.1)	127 (32.6)	< 0.0001
Hemicolectomy or more	561 (51.6)	1631 (57.6)	366 (63.7)	151 (35.9)	380 (22.8)	1064 (60.4)	253 (64.9)	
Other	37 (3.4)	217 (7.7)	42 (7.3)	24 (5.7)	71 (4.3)	79 (4.5)	10 (2.6)	
Chemotherapy								
No	927 (85.3)	1518 (53.6)	207 (36)	400 (95)	1640 (98.4)	1046 (59.4)	219 (56.2)	< 0.0001
Yes	160 (14.7)	1313 (46.4)	368 (64)	21 (5)	27 (1.6)	716 (40.6)	171 (43.9)	
Cancer specific death								
No	968 (89.1)	2135 (75.4)	313 (54.4)	403 (95.7)	1656 (99.3)	1259 (71.5)	290 (74.4)	< 0.0001
Yes	119 (11)	696 (24.6)	262 (45.6)	18 (4.3)	11 (0.7)	503 (28.6)	100 (25.6)	
Overall death								
No	837 (77)	1779 (62.8)	221 (38.4)	372 (88.4)	1605 (96.3)	930 (52.8)	256 (65.6)	< 0.0001
Yes	250 (23)	1052 (37.2)	354 (61.6)	49 (11.6)	62 (3.7)	832 (47.2)	134 (34.4)	

^a^Unmarried status including, divorced, separated, widowed and unmarried. *CI* confidence interval; *HR* hazard ratio; *SRCC* signet ring cell carcinoma; *MAC* mucinous adenocarcinomas; *NMAC* non-mucinous adenocarcinoma; *MiNENs* mixed neuroendocrine non-neuroendocrine neoplasms; *GCC* goblet cell carcinoma; *NETs* neuroendocrine tumors; *NECs*, neuroendocrine carcinomas

**Fig. 1:**
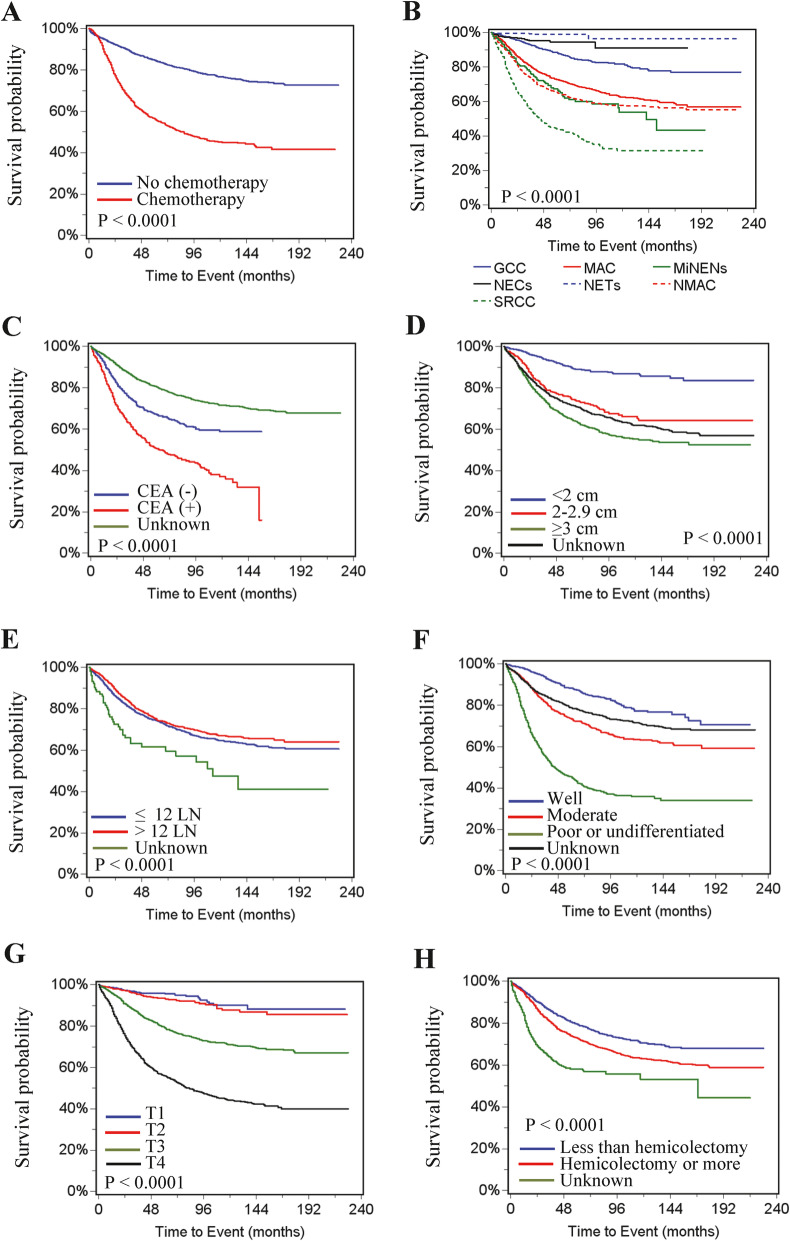
Cancer specific curves for patients with appendiceal cancer. **A** Chemotherapy. **B** Histological types. **C** Serum CEA. **D** Tumor size. **E** Number of lymph node harvested. LN, lymph node. **F** Grade. **G** T stage. **H** Surgery

Univariate survival analysis showed that chemotherapy was significantly associated with worse cancer-specific survival (Hazard ration (HR) =3.54, 95% confidence interval (CI) =3.14–3.99, *P* < 0.0001) (Table 3 and Fig. 1A). In addition, histology of SRCC, MAC and NMAC (vs GCC), older age, unmarried status, African American race, South or Midwest region, positive serum CEA, tumor deposit, tumor size ≥2 cm, advanced T, N, M stages, ≤12 lymph node harvested, higher grade and extent of surgical intervention less than hemicolectomy were associated with reduced cancer-specific survival (Fig. 1B-H). Multivariate analysis revealed that chemotherapy was not associated with cancer-specific survival (HR = 0.93, 95% CI =0.81–1.07, *P* = 0.2983). Compared with patients with GCC, patients with NMAC (HR = 2.26, 95% CI = 1.71–42.99, *P* < 0.0001), SRCC (HR = 1.89, 95% CI = 1.42–2.55, *P* < 0.0001) and MiNENs (HR = 1.72, 95% CI = 1.23–2.41, *P* < 0.0001) had significantly lower cancer-specific survival, NETs (HR = 0.32, 95% CI =0.16–0.65, *P* < 0.0001) had significantly improved cancer-specific survival, and MAC (HR = 1.31, 95% CI =0.99–1.74, *P* = 0.0609) and NECs (HR = 0.76, 95% CI =0.42–1.4, *P* = 0.3766) showed no significant difference. Increased age, unmarried status, African American race, positive serum CEA, tumor deposit, ≤ 12 lymph node harvested, advanced T stage, lymph node metastasis, distant metastasis, and higher grade, were significantly associated with lower cancer-specific survival (Table 3).

**Table 3:** Risk factors correlated with cancer-specific survival in all appendiceal cancer patients

Variable	Cancer-specific Survival				Overall survival			
	Univariate	*P* value	Multivariate	*P* value	Univariate	*P* value	Multivariate	*P* value
	HR (95% CI)		HR (95% CI)		HR (95% CI)		HR (95% CI)	
Age								
≤ 56	1		1		1		1	
> 56	1.46 (1.3–1.64)	<.0001	1.19 (1.05–1.34)	0.0054	1.99 (1.8–2.19)	<.0001	1.61 (1.45–1.78)	<.0001
Gender								
Male	1				1		1	
Female	1.06 (0.94–1.19)	0.3459			0.92 (0.84–1.01)	0.0715	0.82 (0.74–0.9)	<.0001
Marital status								
Married	1		1		1		1	
Unmarried^a^	1.04 (0.92–1.18)	0.507	1.24 (1.09–1.4)	0.0008	1.17 (1.07–1.29)	0.0011	1.35 (1.22–1.49)	<.0001
Unknown	0.78 (0.58–1.06)	0.1106	1.3 (0.96–1.77)	0.092	0.84 (0.66–1.07)	0.1632	1.28 (1–1.63)	0.0501
Race								
African American	1		1		1		1	
White	0.72 (0.6–0.85)	0.0002	0.75 (0.62–0.9)	0.0017	0.75 (0.65–0.86)	<.0001	0.82 (0.71–0.95)	0.0068
Other	0.9 (0.69–1.18)	0.447	0.77 (0.58–1.02)	0.0728	0.82 (0.65–1.02)	0.0744	0.8 (0.63–1.02)	0.0657
Unknown	0.16 (0.04–0.64)	0.0098	0.39 (0.1–1.6)	0.1928	0.16 (0.05–0.49)	0.0014	0.33 (0.11–1.04)	0.0592
Region								
West	1		1		1		1	
South	1.16 (1.01–1.34)	0.0397	1.22 (1.05–1.42)	0.01	1.16 (1.03–1.3)	0.0124	1.2 (1.06–1.35)	0.0034
Midwest	1.38 (1.14–1.67)	0.0008	1.22 (1–1.49)	0.0478	1.29 (1.1–1.51)	0.0014	1.18 (1–1.39)	0.0475
Northwest	0.93 (0.79–1.1)	0.3925	1.03 (0.87–1.21)	0.7785	0.98 (0.86–1.12)	0.7558	1.06 (0.93–1.21)	0.3898
CEA								
Negative	1		1		1		1	
Positive	1.82 (1.54–2.15)	<.0001	1.4 (1.17–1.67)	0.0006	1.67 (1.45–1.93)	<.0001	1.33 (1.14–1.54)	0.0002
Unknown	0.53 (0.45–0.61)	<.0001	0.97 (0.83–1.14)	0.7777	0.63 (0.55–0.71)	<.0001	0.99 (0.87–1.13)	0.909
Deposit								
Negative	1		1		1		1	
Positive	4.21 (3.49–5.07)	<.0001	1.31 (1.07–1.6)	0.008	3.36 (2.88–3.92)	<.0001	1.36 (1.15–1.61)	0.0003
Unknown	1.15 (1–1.32)	0.0431	1.37 (1.19–1.58)	<.0001	1.04 (0.93–1.16)	0.4885	1.22 (1.09–1.37)	0.0007
Tumor size								
< 2 cm	1		1		1		1	
2–2.9 cm	3.45 (2.64–4.5)	<.0001	1.23 (0.93–1.62)	0.1461	2.18 (1.8–2.65)	<.0001	1.12 (0.91–1.38)	0.2898
≤ 3 cm	5.2 (4.2–6.42)	<.0001	1.33 (1.06–1.67)	0.0143	3.19 (2.76–3.69)	<.0001	1.25 (1.06–1.47)	0.0085
Unknown	4.53 (3.64–5.65)	<.0001	1.33 (1.06–1.69)	0.0163	2.87 (2.46–3.34)	<.0001	1.24 (1.05–1.47)	0.0132
Harvested lymph nodes								
≤ 12	1		1		1		1	
> 12	0.88 (0.78–0.99)	0.0318	0.58 (0.51–0.66)	<.0001	0.82 (0.75–0.91)	<.0001	0.61 (0.55–0.68)	<.0001
Unknown	1.76 (1.14–2.72)	0.0108	0.85 (0.55–1.32)	0.5551	1.46 (1–2.12)	0.0501	0.82 (0.56–1.2)	0.2969
Histology								
GCC	1		1		1		1	
MAC	2.39 (1.85–3.09)	<.0001	1.31 (0.99–1.74)	0.0609	1.81 (1.5–2.19)	<.0001	1.16 (0.94–1.43)	0.1765
NMAC	3.59 (2.77–4.67)	<.0001	2.26 (1.71–2.99)	<.0001	2.91 (2.4–3.53)	<.0001	2.05 (1.66–2.53)	<.0001
SRCC	6.81 (5.16–8.99)	<.0001	1.89 (1.4–2.55)	<.0001	4.63 (3.75–5.71)	<.0001	1.78 (1.41–2.24)	<.0001
NECs	0.42 (0.23–0.74)	0.0027	0.76 (0.42–1.4)	0.3776	0.63 (0.44–0.89)	0.0093	0.95 (0.65–1.39)	0.7755
NETs	0.12 (0.06–0.23)	<.0001	0.32 (0.16–0.65)	0.0017	0.35 (0.26–0.48)	<.0001	0.64 (0.45–0.92)	0.0171
MiNENs	3.03 (2.18–4.2)	<.0001	1.72 (1.23–2.41)	0.0016	2.08 (1.61–2.7)	<.0001	1.4 (1.07–1.83)	0.0137
T stage								
T1	1		1		1		1	
T2	1.54 (0.96–2.46)	0.0741	0.77 (0.47–1.24)	0.2788	1.34 (1.03–1.76)	0.0327	0.79 (0.59–1.05)	0.1077
T3	4.51 (3.32–6.13)	<.0001	1.51 (1.08–2.12)	0.0165	2.38 (1.99–2.85)	<.0001	1.15 (0.93–1.43)	0.1923
T4	12.72 (9.5–17.02)	<.0001	2.38 (1.71–3.32)	<.0001	5.2 (4.4–6.15)	<.0001	1.69 (1.37–2.09)	<.0001
N stage								
N0	1		1		1		1	
N1	3.14 (2.72–3.62)	<.0001	2.26 (1.93–2.65)	<.0001	2.32 (2.06–2.61)	<.0001	2 (1.75–2.28)	<.0001
N2	8.15 (7.05–9.42)	<.0001	3.03 (2.53–3.62)	<.0001	5.8 (5.14–6.56)	<.0001	2.89 (2.48–3.37)	<.0001
M stage								
M0	1		1		1		1	
M1	5.46 (4.85–6.16)	<.0001	2.45 (2.1–2.86)	<.0001	3.47 (3.16–3.81)	<.0001	2.04 (1.8–2.31)	<.0001
Grade								
Well differentiated	1		1		1		1	
Moderately differentiated	2.83 (2.33–3.44)	<.0001	1.62 (1.32–1.99)	<.0001	2.32 (2.01–2.67)	<.0001	1.49 (1.28–1.73)	<.0001
Poorly or un-differentiated	8.52 (7.08–10.26)	<.0001	2.73 (2.2–3.39)	<.0001	5.44 (4.73–6.25)	<.0001	2.21 (1.87–2.62)	<.0001
Unknown	2.53 (2.05–3.11)	<.0001	2.16 (1.73–2.69)	<.0001	2.08 (1.78–2.42)	<.0001	1.71 (1.45–2.02)	<.0001
Surgery								
Less than hemicolectomy	1				1		1	
Hemicolectomy or more	1.43 (1.26–1.63)	<.0001			1.22 (1.11–1.35)	<.0001	0.89 (0.8–0.98)	0.024
Other	2.88 (2.36–3.52)	<.0001			2.16 (1.82–2.56)	<.0001	1.51 (1.26–1.81)	<.0001
Chemotherapy								
No	1		1		1		1	
Yes	3.54 (3.14–3.99)	<.0001	0.93 (0.8–1.07)	0.2983	2.11 (1.93–2.32)	<.0001	0.73 (0.65–0.82)	<.0001

^a^Unmarried status including divorced, separated, widowed and unmarried. *CI* confidence interval; *HR* hazard ratio; *SRCC* signet ring cell carcinoma; *MAC* mucinous adenocarcinomas; *NMAC* non-mucinous adenocarcinoma; *MiNENs* mixed neuroendocrine non-neuroendocrine neoplasms; *GCC* goblet cell carcinoma; *NETs* neuroendocrine tumors; *NECs* neuroendocrine carcinomas

**Fig. 2:**
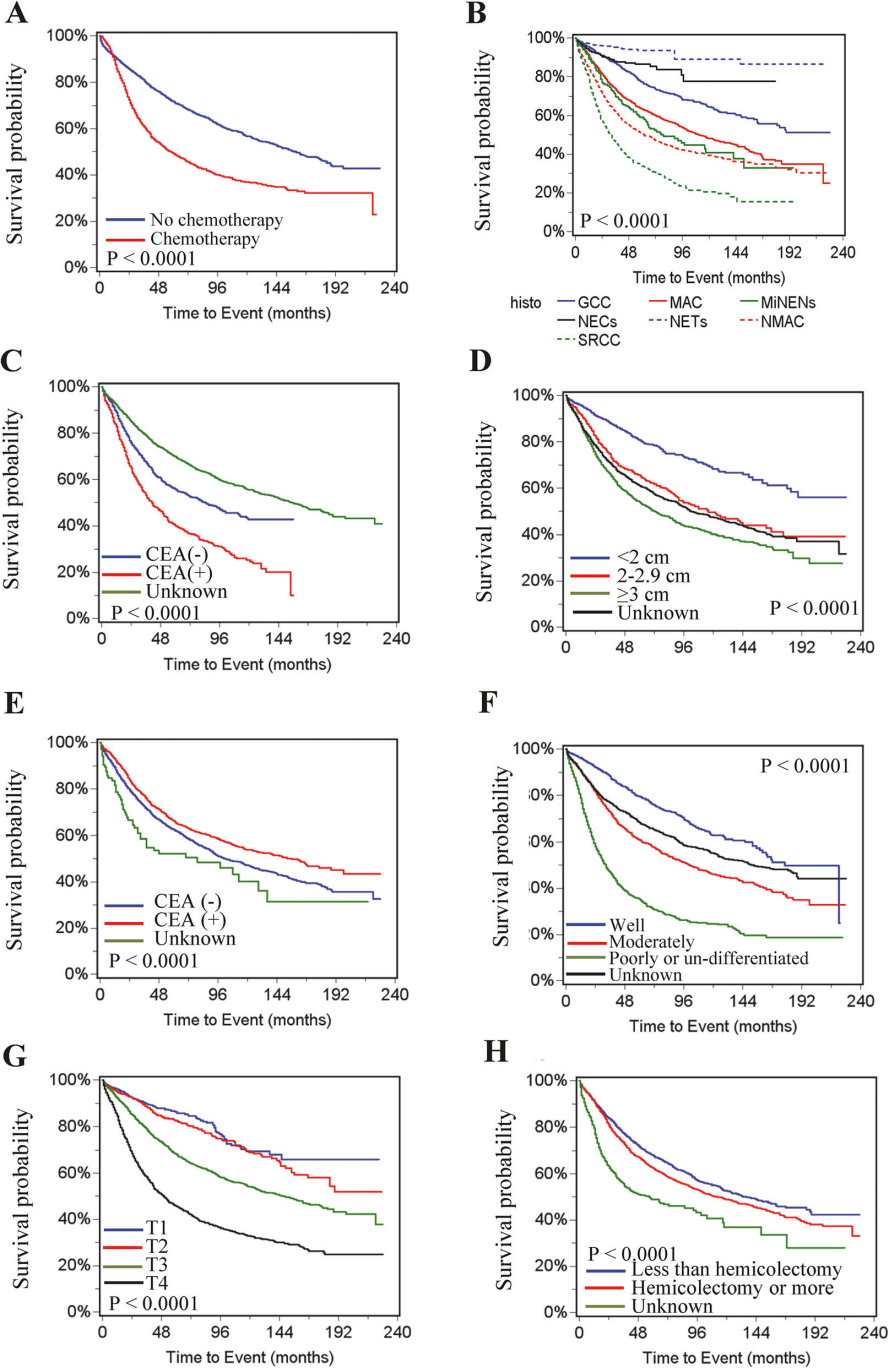
Overall survival for patients with appendiceal cancer. **A** Chemotherapy. **B** Histological types. **C** Serum CEA. **D** Tumor size. **E** Number of lymph node harvested. LN, lymph node. **F** Grade. **G** T stage. **H** Surgery

Our data showed that chemotherapy was significantly associated with worse overall survival (HR = 2.11, 95% CI =1.93–2.32, *P* < 0.0001) in a univariate analysis (Table 3**and** Fig. 2A). Similarly, histology of SRCC, MAC and NMAC, increased age, male, unmarried status, African American race, South or Midwest region, positive serum CEA, tumor deposit, ≤12 lymph node harvested, T3 or T4 stage, lymph node metastasis, distant metastasis, higher grade and less than hemicolectomy were significantly associated with worse overall survival (Fig. 2B-H). Multivariate analysis showed chemotherapy was significantly associated with improved overall survival (HR = 0.73, 95% CI =0.65–0.82, *P* < 0.0001). Patients with MiNENs, NMAC and SRCC had significantly worse overall survival than patients with GCC, whereas patients with MAC, NECs and NETs had no significant difference in overall survival compared to patients with GCC. Patients with increased age, unmarried status, African American race, positive serum CEA, ≤12 harvested lymph nodes, T4 stage, lymph node metastasis, distant metastasis and higher grade were all significantly associated with worse overall survival in both univariate and multivariate analysis (Table 3).

Univariate analysis indicated that chemotherapy was significantly associated with worse cancer specific and overall survival during 1998–2011 (Supplemental data Fig. S1A-B) and 2012–2016 (Supplemental data Fig. S1C-D). Multivariate analysis showed that chemotherapy was not significantly associated with cancer-specific survival (HR = 1.1, 95% CI =0.92–1.33, *P* = 0.2937), but was significantly associated with improved overall survival (HR = 0.86, 95% CI =0.74–0.99, *P* = 0.0385) in all patients diagnosed during 1998–2011 (Supplemental data Table S1). In contrast, chemotherapy significantly improved both cancer-specific survival (HR = 0.72, 95% CI =0.58–0.91, *P* < 0.0001) and overall survival (HR = 0.59, 95% CI =0.49–0.71, *P* < 0.0001) in all patients diagnosed during 2012–2016 (Table 4).

**Table 4:** Risk factors correlated with cancer-specific and overall survival in all appendiceal cancer patients diagnosed during 2012–2016

Variable	Cancer-specific survival		Overall survival	
	HR (95% CI)	*P* value	HR (95% CI)	*P* value
Age				
≤ 56			1	
> 56			1.63 (1.39–1.92)	<.0001
Sex				
Male			1	
Female			0.83 (0.71–0.96)	0.015
Marital status				
Married	1		1	
Unmarried^a^	1.24 (1.02–1.5)	0.03	1.24 (1.06–1.45)	0.0065
Unknown	1.09 (0.66–1.8)	0.7293	1.18 (0.82–1.71)	0.3771
Region				
West			1	
South			1.37 (1.14–1.64)	0.0006
Midwest			1.25 (0.97–1.61)	0.0847
Northwest			1.19 (0.96–1.47)	0.1055
CEA				
Negative	1		1	
Positive	1.49 (1.13–1.96)	0.0052	1.3 (1.02–1.64)	0.0316
Unknown	0.91 (0.71–1.19)	0.4996	0.95 (0.77–1.18)	0.6546
Deposit				
Negative	1		1	
Positive	1.45 (1.11–1.9)	0.0064	1.53 (1.22–1.91)	0.0002
Unknown	1.44 (1.15–1.8)	0.0013	1.23 (1.02–1.48)	0.027
Harvested lymph nodes	1			
≤ 12	0.56 (0.46–0.69)	<.0001		
> 12	1.12 (0.49–2.57)	0.7879		
Unknown				
Histology				
GCC	1		1	
MAC	1.8 (1.17–2.78)	0.0075	1.6 (1.16–2.22)	0.0046
NMAC	2.76 (1.81–4.21)	<.0001	2.56 (1.86–3.5)	<.0001
SRCC	2.21 (1.39–3.52)	0.0009	2.18 (1.53–3.12)	<.0001
MiNENs	2.19 (1.32–3.66)	0.0026	1.67 (1.11–2.52)	0.0136
NECs	0.54 (0.19–1.5)	0.235	0.99 (0.54–1.81)	0.9704
NETs	0.3 (0.12–0.73)	0.0086	0.89 (0.54–1.46)	0.6515
T stage				
T1	1		1	
T2	0.73 (0.34–1.57)	0.4247	0.95 (0.6–1.49)	0.8078
T3	1.14 (0.65–1.99)	0.642	1.21 (0.83–1.77)	0.322
T4	1.71 (1–2.94)	0.0516	1.73 (1.19–2.51)	0.0042
N stage				
N0	1		1	
N1	2.46 (1.91–3.17)	<.0001	0.56 (0.48–0.66)	<.0001
N2	3.73 (2.84–4.9)	<.0001	1.18 (0.62–2.24)	0.6124
M stage				
M0	1		1	
M1	2.54 (1.98–3.26)	<.0001	2.15 (1.76–2.62)	<.0001
Grade				
Well differentiated	1		1	
Moderately differentiated	2.38 (1.66–3.42)	<.0001	1.84 (1.44–2.36)	<.0001
Poorly or un-differentiated	3.4 (2.32–4.97)	<.0001	2.46 (1.88–3.23)	<.0001
Unknown	3.2 (2.18–4.71)	<.0001	2.42 (1.85–3.15)	<.0001
Chemotherapy				
No	1		1	
Yes	0.72 (0.58–0.91)	0.0051	0.59 (0.49–0.71)	<.0001

^a^Unmarried status, including divorced, separated, widowed and unmarried. *CI* confidence interval; *HR* hazard ratio; *SRCC* signet ring cell carcinoma; *MAC*, mucinous adenocarcinomas; *NMAC* non-mucinous adenocarcinoma; *MiNENs* mixed neuroendocrine non-neuroendocrine neoplasms; *GCC* goblet cell carcinoma; *NETs* neuroendocrine tumors; *NECs* neuroendocrine carcinomas

**Fig. 3:**
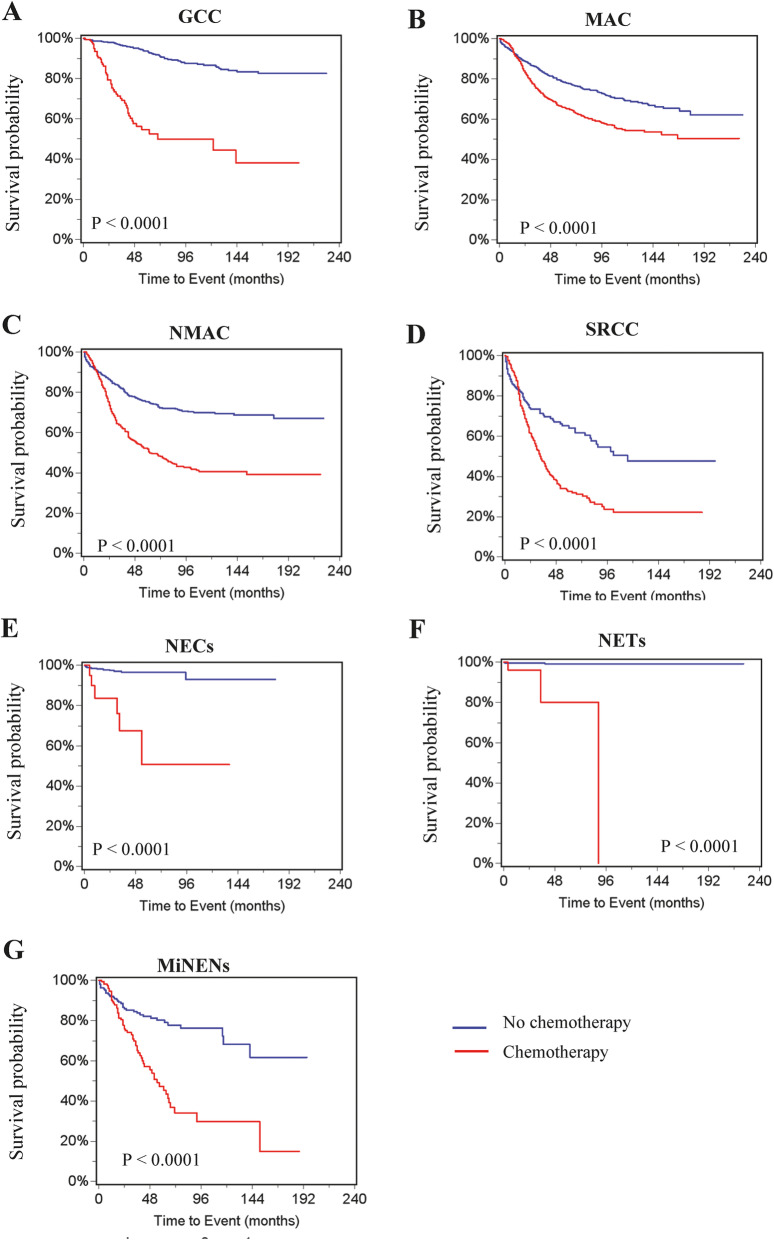
Cancer specific curves for patients with different histological type of appendiceal cancer. **A** GCC. **B** MAC. **C** NMAC. **D** SRCC. **E** NECs. **F** NETs. **G** MiNENS

**Fig. 4:**
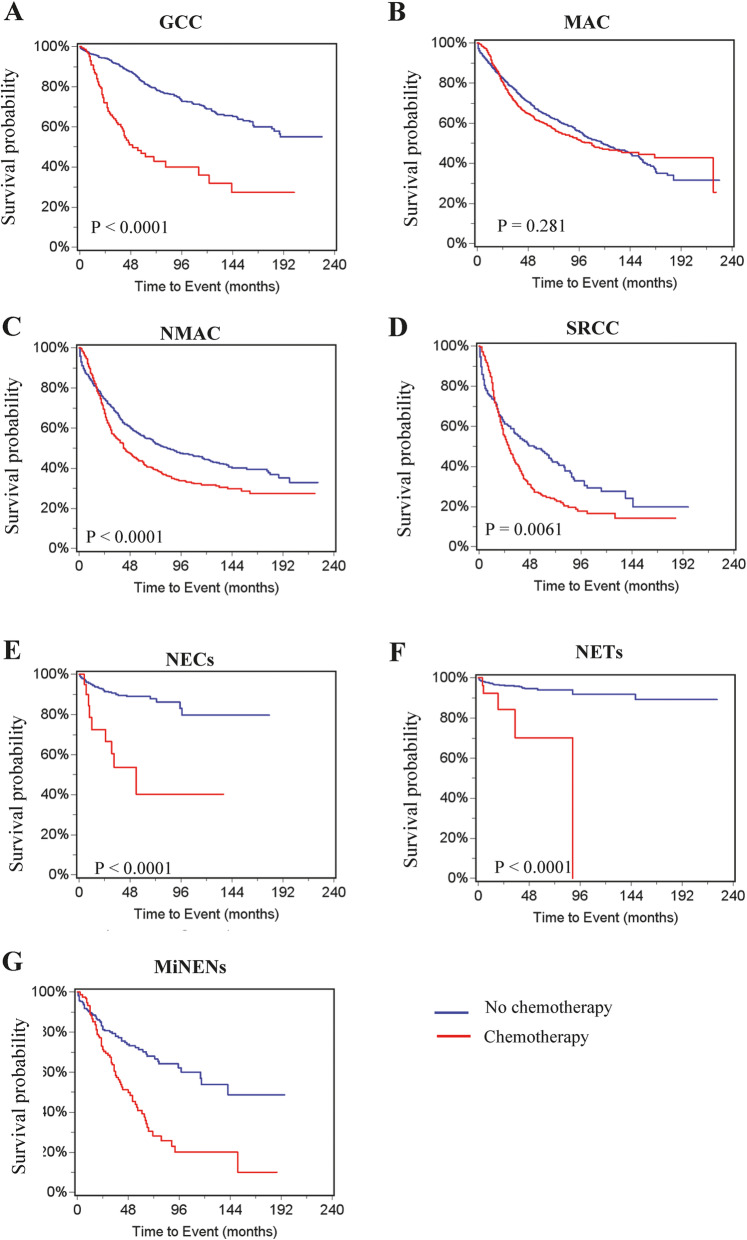
Overall survival for patients with different histological type of appendiceal cancer. **A** GCC. **B** MAC. **C** NMAC. **D** SRCC. **E** NECs. **F** NETs. **G** MiNENS

We then determined the effect of chemotherapy in the treatment of individual histological types. Survival rates at 5 and 10 years were compared for patients that did and did not undergo chemotherapy (Table 5**)**. Our results revealed that chemotherapy was significantly associated with lower cancer-specific survival in all histological types (Fig. 3A-G), and overall survival in all histologically types except MAC (Fig. 4A-G). Very few patients with NETs or NECs received chemotherapy and died from the disease.

**Table 5:** Comparison of survival rates between patients treated with or without chemotherapy in appendiceal cancer of different histological types

	Cancer-specific death					Overall death				
	Case (%)	Deaths	5 years	10 years	*P* value	Case (%)	Deaths	5 years	10 years	*P* value
GCC										
No chemotherapy	927 (85.3)	68	0.9364	0.8669	<.0001	927 (85.3)	183	0.8255	0.6896	<.0001
Chemotherapy	160 (14.7)	51	0.5455	0.4994		160 (14.7)	67	0.4678	0.3588	
MAC										
No chemotherapy	1518 (53.6)	313	0.7876	0.6911	<.0001	1518 (53.6)	582	0.6584	0.4944	0.281
Chemotherapy	1313 (46.4)	383	0.6599	0.5438		1313 (46.4)	470	0.6078	0.4714	
NMAC										
No chemotherapy	1046 (59.4)	217	0.7531	0.6998	<.0001	1046 (59.4)	461	0.5621	0.4454	<.0001
Chemotherapy	716 (40.6)	286	0.5231	0.4072		716 (40.6)	371	0.4375	0.318	
SRCC										
No chemotherapy	207 (36)	68	0.6508	0.4773	<.0001	207 (36)	119	0.4819	0.2768	<.0001
Chemotherapy	368 (64)	194	0.3274	0.2215		368 (64)	235	0.2574	0.166	
NECs										
No chemotherapy	400 (95)	12	0.9662	0.9304	<.0001	400 (95)	40	0.8896	0.7974	<.0001
Chemotherapy	21 (5)	6	0.5071	0.5071		21 (5)	9	0.4031	0.4031	
NETs										
No chemotherapy	1640 (98.4)	8	0.8013		<.0001	1640 (98.4)	57	0.9401	0.9177	<.0001
Chemotherapy	27 (1.6)	3	0.8013			27 (1.6)	5	0.7017		
MiNENs										
No chemotherapy	219 (56.2)	39	0.8021	0.6821	<.0001	219 (56.2)	69	0.7125	0.5378	<.0001
Chemotherapy	171 (43.9)	61	0.4726	0.2989		171 (43.9)	75	0.4092	0.202

Multivariate analysis showed that chemotherapy was significantly associated with cancer specific in patients with NMAC only during 2012–2016 (HR = 0.66, 95% CI =0.44–0.99, *P* = 0.0449), MAC (HR = 0.49, 95% CI =0.31–0.77, *P* = 0.0021) only during 2009–2016 and SRCC (HR = 0.23, 95% CI =0.11–0.50, *P* = 0.0002) only during 2013–2016. In contrast, there was no significant association between chemotherapy and cancer specific survival in patients with GCC or MiNENs (Table 6**)** and in patient with NETs or NECs (Supplemental data Table S2). Number (> 12) of sampled lymph nodes was significantly associated with improved cancer specific survival in patients with NMAC, MAC, and SRCC. Other prognostic factors were also identified to be associated with cancer specific survival in patients with individual histological type.

**Table 6:** Risk factors correlated with cancer specific in patients with individual histological type of appendiceal cancer

Variable										
Study period	2012–1016		2009–2016		2013–2016		2009–2016		1998–2016	
Histological type	NMAC		MAC		SRCC		GCC		MiNENs	
	HR (95% CI)	*P* value	HR (95% CI)	*P* value	HR (95% CI)	*P* value	HR (95% CI)	*P* value	HR (95% CI)	*P* value
Marital status										
Married^a^			1							
Unmarried			1.49 (1.18–1.87)	0.0006						
Unknown			1.06 (0.57–1.97)	0.8627						
Race										
African American							1			
White							0.22 (0.12–0.41)	<.0001		
Other							0.09 (0.02–0.49)	0.0053		
Unknown							0	0.9866		
Region										
West			1							
South			1.22 (0.92–1.62)	0.1647						
Midwest			1.47 (1.01–2.14)	0.0428						
Northwest			1.01 (0.72–1.4)	0.9737						
CEA										
Negative	1				1					
Positive	1.86 (1.15–3)	0.0113			2.51 (1.4–4.49)	0.002				
Unknown	1.22 (0.77–1.93)	0.3881			1.5 (0.91–2.45)	0.1092				
Deposit										
Negative	1		1				1			
Positive	1.75 (1.09–2.82)	0.0209	1.99 (1.41–2.82)	<.0001			2.57 (1.07–6.17)	0.0346		
Unknown	1.55 (1.04–2.3)	0.0296	1.4 (1.09–1.81)	0.0095			2.04 (1.16–3.57)	0.0133		
Tumor size										
< 2 cm							1			
2–2.9 cm							2.28 (0.75–6.97)	0.1468		
≤ 3 cm							3.14 (1.16–8.51)	0.0242		
Unknown							2.82 (1–7.96)	0.0502		
Harvested lymph nodes										
≤ 12	1		1		1					
> 12	0.62 (0.42–0.91)	0.0143	0.66 (0.52–0.84)	0.0006	0.59 (0.39–0.89)	0.0116				
Unknown	1.1 (0.33–3.71)	0.8738	0.54 (0.22–1.33)	0.1788	0	0.9885				
T stage										
T1			1						0.64 (0.08–4.85)	0.6656
T2			0.94 (0.33–2.72)	0.9087					0.8 (0.11–6.07)	0.8308
T3			1.67 (0.86–3.25)	0.1277					1	
T4			2.57 (1.39–4.76)	0.0027					2.59 (1.43–4.66)	0.0016
N stage										
N0	1		1		1		1			
N1	2.01 (1.31–3.08)	0.0015	2.4 (1.76–3.28)	<.0001	2.48 (1.46–4.22)	0.0008	2.67 (1.28–5.57)	0.0088		
N2	2.69 (1.66–4.38)	<.0001	3.81 (2.61–5.55)	<.0001	3.23 (1.94–5.39)	<.0001	5.65 (2.38–13.41)	<.0001		
M stage										
M0	1		1		1	1	1		1	
M1	3.02 (1.97–4.63)	<.0001	0.55 (0.42–0.72)	<.0001	2.68 (1.65–4.35)	<.0001	6.89 (3.2–14.82)	<.0001	3.59 (2.05–6.3)	<.0001
Grade										
Well differentiated	1		1							
Moderately differentiated	3.41 (1.43–8.09)	0.0055	1.81 (1.35–2.43)	<.0001						
Poorly or un-differentiated	4.81 (1.97–11.74)	0.0006	3.05 (2.12–4.39)	<.0001						
Unknown	2.87 (1.1–7.5)	0.0315	2.41 (1.69–3.43)	<.0001						
Surgery										
Less than hemicolectomy	1									
Hemicolectomy or more	0.75 (0.51–1.11)	0.1493								
Other	2.72 (1.39–5.35)	0.0036								
Chemotherapy										
No	1		1		1		1		1	
Yes	0.67 (0.45–1)	0.0493	0.78 (0.6–0.997)	0.0477	0.54 (0.33–0.89)	0.0151	2.0 (0.94–4.28)	0.0734	0.78 (0.43–1.4)	0.4029

^a^Unmarried status, including divorced, separated, widowed and unmarried. *CI* confidence interval; *HR* hazard ratio; *SRCC* signet ring cell carcinoma; *MAC* mucinous adenocarcinomas; *NMAC* non-mucinous adenocarcinoma; *MiNENs* mixed neuroendocrine non-neuroendocrine neoplasms; *GCC* goblet cell carcinoma; *NETs* neuroendocrine tumors; *NECs* neuroendocrine carcinomas

Multivariate survival analysis revealed that chemotherapy was significantly associated with overall survival in patients with MAC (HR = 0.72, 95% CI =0.61–0.86, *P* < 0.0001), NMAC (HR = 0.72, 95% CI =0.61–0.86, *P* = 0.0003) and SRCC (HR = 0.62, 95% CI =0.46–0.84, P = 0.0002) during the whole study period. In contrast, there was no significant association between chemotherapy and overall survival in patients with GCC, MiNENs (Table 7**),** NETs and NECs (Supplemental data Table S2). Number (> 12) of harvested lymph nodes was one of the prognostic factors associated with better overall survival in patients with GCC, NMAC, MAC, SRCC and MiNENs. Other prognostic factors associated with overall survival were identified in patients with different histological type.

**Table 7:** Risk factors correlated with overall survival in patients with specific histological types of appendiceal cancer

Variable	MAC		NMAC		SRCC		GCC		MiNENs	
	HR (95% CI)	*P* value	HR (95% CI)	*P* value	HR (95% CI)	*P* value	HR (95% CI)	*P* value	HR (95% CI)	*P* value
Age										
≤ 56	1		1		1		1		1	
> 56	1.44 (1.22–1.69)	<.0001	1.45 (1.2–1.76)	0.0001	1.32 (1.02–1.71)	0.0381	1.6 (1.1–2.31)	0.0128	1.78 (1.14–2.79)	0.0116
Gender										
Male	1									
Female	0.71 (0.6–0.83)	<.0001								
Marital status										
Married	1		1						1	
Unmarried^a^	1.41 (1.19–1.66)	<.0001	1.51 (1.26–1.8)	<.0001					2.12 (1.33–3.37)	0.0015
Unknown	1.18 (0.75–1.85)	0.4711	1.42 (0.93–2.17)	0.1067					1.15 (0.44–3.02)	0.7828
Race										
African American							1			
White							0.44 (0.27–0.71)	0.0008		
Other							0.15 (0.03–0.65)	0.0114		
Unknown							0!	0.9752		
Region										
West	1		1							
South	1.25 (1.03–1.52)	0.0224	1.39 (1.13–1.71)	0.0021						
Midwest	1.49 (1.15–1.93)	0.0027	1.14 (0.83–1.57)	0.4065						
Northwest	1.02 (0.81–1.28)	0.886	1.08 (0.84–1.38)	0.5372						
CEA										
Negative	1		1		1					
Positive	1.36 (1.06–1.75)	0.0168	1.49 (1.14–1.95)	0.0035	1.62 (1.13–2.32)	0.009				
Unknown	0.89 (0.7–1.12)	0.3117	1.27 (1–1.6)	0.046	1.12 (0.83–1.52)	0.4668				
Deposit										
Negative	1		1				1			
Positive	1.75 (1.31–2.33)	0.0001	1.38 (1.02–1.86)	0.0365			2.59 (1.29–5.19)	0.0074		
Unknown	1.15 (0.95–1.38)	0.1512	1.32 (1.08–1.61)	0.0059			1.25 (0.86–1.82)	0.2347		
Harvested lymph nodes										
≤ 12	1		1		1		1		1	
> 12	0.64 (0.54–0.75)	<.0001	0.6 (0.49–0.73)	<.0001	0.65 (0.49–0.85)	0.0016	0.51 (0.34–0.77)	0.0015	0.31 (0.19–0.49)	<.0001
Unknown	0.59 (0.32–1.08)	0.0866	1.27 (0.66–2.42)	0.4734	1.25 (0.3–5.22)	0.761	4.46 (0.56–35.46)	0.1572	1.97 (0.44–8.92)	0.3789
T stage										
T1	1		1	1	1.74 (0.76–3.96)	0.189	0.65 (0.32–1.33)	0.2409	0.74 (0.17–3.15)	0.6795
T2	1.21 (0.72–2.03)	0.4692	0.97 (0.67–1.41)	0.8767	1.42 (0.5–4.04)	0.5097	0.32 (0.12–0.88)	0.0267	0.77 (0.1–6.08)	0.7997
T3	1.47 (1.03–2.1)	0.0363	0.58 (0.4–0.83)	0.0027	1		1		1	
T4	1.78 (1.27–2.49)	0.0009	1.45 (1.18–1.79)	0.0005	2.53 (1.74–3.69)	<.0001	1.08 (0.67–1.74)	0.7398	2.1 (1.26–3.51)	0.0046
N stage										
N0	1		1		1		1		1	
N1	2.09 (1.66–2.61)	<.0001	1.97 (1.57–2.47)	<.0001	1.74 (1.23–2.46)	0.0016	2.28 (1.28–4.05)	0.0052	2.38 (1.26–4.49)	0.0075
N2	3.3 (2.47–4.4)	<.0001	2.66 (2.03–3.47)	<.0001	2.3 (1.65–3.2)	<.0001	4.39 (2.26–8.53)	<.0001	5.66 (2.98–10.73)	<.0001
M stage										
M0	1		1		1		1		1	
M1	0.63 (0.52–0.76)	<.0001	2.56 (2.02–3.26)	<.0001	0.46 (0.33–0.63)	<.0001	0.31 (0.17–0.58)	0.0002	0.32 (0.19–0.52)	<.0001
Grade										
Well differentiated	1		1		0.65 (0.13–3.23)	0.593			1	
Moderately differentiated	1.58 (1.3–1.92)	<.0001	1.48 (1.06–2.07)	0.0205	1				2.21 (0.61–7.93)	0.2253
Poorly or un-differentiated	2.28 (1.75–2.96)	<.0001	1.96 (1.38–2.79)	0.0002	2.67 (1.17–6.09)	0.0192			4.26 (1.41–12.88)	0.0102
Unknown	1.65 (1.3–2.09)	<.0001	1.93 (1.27–2.92)	0.0021	3.25 (1.39–7.59)	0.0065			2.99 (0.97–9.21)	0.0569
Surgery										
Less than hemicolectomy			1				1			
Hemicolectomy or more			0.71 (0.59–0.86)	0.0004			0.65 (0.44–0.96)	0.0292		
Other			1.7 (1.19–2.41)	0.0034			1.95 (0.97–3.96)	0.0628		
Chemotherapy										
No	1		1		1		1		1	
Yes	0.72 (0.61–0.86)	0.0003	0.61 (0.49–0.76)	<.0001	0.62 (0.46–0.84)	0.002	1.29 (0.76–2.19)	0.352	1.07 (0.63–1.8)	0.8081

^a^Unmarried status, including divorced, separated, widowed and unmarried. *CI* confidence interval; *HR* hazard ratio; *SRCC* signet ring cell carcinoma; *MAC* mucinous adenocarcinomas; *NMAC* non-mucinous adenocarcinoma; *MiNENs* mixed neuroendocrine non-neuroendocrine neoplasms; *GCC* goblet cell carcinoma; *NETs* neuroendocrine tumors; *NECs* neuroendocrine carcinomas

## Discussion

This study examined the use of chemotherapy and its potential association with survival in patients with different histological types of appendiceal cancer using the SEER database. The results revealed that chemotherapy was administrated at highly varied rates among different histological types. Chemotherapy significantly improved cancer-specific survival in all patients diagnosed during 2012–2016, and in patients with NMAC during 2012–2016, MAC during 2009–2016 and SRCC during 2013–2016, though chemotherapy was significantly associated with overall survival in the entire study period. This finding suggests that chemotherapy provides survival benefits in the treatment of appendiceal cancer on the whole, and particularly for certain histological types. The efficacy of chemotherapy in the treatment of these cancers appears to have improved in recent years.

This study found that MAC and NMAC were the most common histological types in all cases examined in this study. Our results revealed that chemotherapy was significantly associated with both improved cancer-specific survival in recent years or overall survival in patients with MAC and NMAC appendiceal cancer. The beneficial effect of chemotherapy in treatment of appendiceal adenocarcinoma has been reported in previous studies. Using the National Cancer Data Base (NCDB), a retrospective study that included a total of 11,871 appendiceal cancer patients diagnosed during 1985 and 2006 was carried out. Only the overall survival information was available in the database. Multivariate analysis showed that chemotherapy improved overall survival for both MAC and NMAC in stage I to III disease. For patients with stage IV disease, chemotherapy significantly improved overall survival for those with NMAC, but not MAC [5]. Another study reported on 109 metastatic NMAC appendiceal cancer patients treated with chemotherapy. Patients who received combination chemotherapy (either oxaliplatin or irinotecan-based) had significantly improved overall survival compared to those receiving fluoropyrimidine monotherapy,  and patients with moderately and poorly differentiated tumors had similar outcomes [9]. Kolla et al. recently reported that in a study of 103 patients with appendiceal adenocarcinoma, adjuvant chemotherapy following complete cytoreduction significantly improved overall survival compared to cytoreduction alone [12]. In contrast, other studies reported no beneficial effect of chemotherapy in the treatment of appendiceal adenocarcinoma [6, 13, 14].

Although SRCC cases only accounted for 6.6% of all examined appendiceal cancers in this study, these patients were among the highest proportion diagnosed at T4 stage (61.4%) and treated with chemotherapy (60%). Patients with SRCC had significantly lower survival than other histological types. Multivariate analysis showed that chemotherapy was significantly associated improved cancer-specific survival in SRCC patients diagnosed during 2013–2016 and overall survival in patients in the entire study period. Our studies indicate that lymph node or distant metastasis, advanced stage and positive serum CEA were associated with significantly reduced cancer-specific survival, whereas patients that had > 12 harvested lymph nodes harvested showed improved cancer specific survival. Based upon this finding, chemotherapy and harvesting of more than 12 lymph nodes are strongly recommended in the treatment of these patients. A retrospective study reported systemic chemotherapy had a survival benefit in patients who were suboptimal candidates for cytoreductive surgery [7]. Another retrospective study reported that poorly differentiated or SRCC appendiceal cancer patients who responded to chemotherapy had improved progression-free survival [8]. However, Barrak et al. reported no survival effect of chemotherapy in the treatment of patients with stage IV appendiceal cancers including SRCC [10].

In this study, 1667 (19.2%) NETs and 421 (4.3%) NECs were identified from the SEER database. Most of these cancers were diagnosed at younger ages and at early stages with smaller tumor sizes. Consistent with previous studies, patients with neuroendocrine appendiceal cancers were diagnosed at a much younger age than patients with other primary appendiceal cancers [15, 16]. Serum CEA is a commonly examined serum marker in the diagnosis of gastrointestinal cancer patients. However, this marker was examined in very few patients with NETs and NECs. This study found that a high proportion of patients had tumors that were less than 2 cm in size. As a result, these patients had the lowest percentages of distant metastasis or lymph node metastasis. A much lower proportion of these patients were treated with chemotherapy. Taken together, these results imply that most of these cancers were detected incidentally, during histopathological examination of the appendix [17]. Multivariate analysis indicated that these cancers were associated with higher rates of survival, compared to appendiceal cancers of other histological types. Chemotherapy was not significantly associated with cancer-specific or overall survival.  Because few patients were treated with chemotherapy and few deaths occurred in these patients, studies with larger sample sizes are needed to study the effect of chemotherapy on the treatment of these histological types of appendiceal cancer.

Appendiceal goblet cell carcinoid (GCC) is a histological type with characteristic goblet cells mixed with neuroendocrine tumors [18, 19]. In this study, a total number of 1087 (12.5%) of GCC patients were identified. Among this group, 119 (11%) patients died of this cancer and 250 (23%) died from all causes. Univariate analysis showed that chemotherapy was associated with lower cancer-specific and overall survival. Multivariate analysis revealed that GCC patients had worse prognoses than classic NECs and NETs patients [20], but a better survival rate than some other histological types. Multivariate analysis of survival rates showed chemotherapy was not significantly associated with cancer-specific survival in patients with GCC during 2009–2016, or overall survival in patients with GCC during 1998–2016. Sporadic studies previously reported that chemotherapy didn’t significantly improve survival in patients with GCC appendiceal cancer [18, 21–23]. Both North American Neuroendocrine Tumor Society (NANETS) and the European Neuroendocrine Tumor Society (ENETS) recommend hemicolectomy as the primary therapy for resectable appendiceal GCC [24]. This study found that hemicolectomy or more extensive surgery was significantly associated with improved survival in patients with GCC. Recent studies reported that adjuvant chemotherapy significantly improved overall survival in a specific group of patients, such as including those with lymph node-positive GCC [25] or stage III GCC after hemicolectomy [26].

MiNEN, previously also *referred to* as *mixed adenoneuroendocrine carcinoma (MANEC*), is another rare histological subtype of appendiceal cancer. It is a hybrid tumor comprised of both neuroendocrine and non-neuroendocrine adenocarcinoma components. In this study, only 390 (4.5%) patients were given a diagnosis of MiNENs, but 100 (25.6%) died of this cancer and 134 (34.4%) died from all causes. Consistent with findings in previous studies [2, 27], this study found that patients with MiNENs had a significantly lower survival rate than GCC patients. Multivariate analyses demonstrated that T4 stage and distant metastasis were significantly associated with cancer-specific survival, while increased age, unmarried status, T4 stage, lymph node, distant metastasis and higher grade were significantly associated with worse overall survival in patients with MiNENs appendiceal cancer. However, chemotherapy was not significantly associated with cancer-specific or overall survival.

This study showed that lymph node metastasis was an independent prognostic biomarker associated with lower cancer-specific survival in all histological types except MiNENS and NETs. and overall survival in all histological types except NETs. Lymph node metastasis has not been associated with lower survival in patients with MAC [28]. Interestingly, this study found that cleaning > 12 lymph nodes was associated with improved cancer-specific survival and overall survival in all patients combined, or in NMAC, MAC, SRCC and GCC, but only with improved overall survival in patients with MiNENs. In colorectal cancer, cleaning at least 12 lymph nodes for adequate staging is recommended by National Comprehensive Cancer Network (NCCN) guidelines [29], and has been associated with better prognosis [30, 31]. Our results suggest that adequate lymph node cleaning may improve survival in some appendiceal cancer patients.

There are several limitations in this study. It was a retrospective study, and there may have been a bias in patient selection that was not controlled for. Due to the complex histological types of the disease, misclassification of certain histological types was likely, particularly for NETs and NECs. Detailed information was missing from the database, such as drugs, dose and duration used in chemotherapy. The intents (palliative or curative) of chemotherapy were not described in the database. It was unknown whether chemotherapy was provided before or after the surgery, or both. The database lacked important data related to prognosis, such as patients’ performance, nutritional status, side effects of chemotherapy and post-operative complications. Cancer markers and other clinical outcomes, such as the radiographic response and recurrence, which were useful in the evaluation of the efficacy of chemotherapy, were also not available in the database.

## Conclusions

The rates at which chemotherapy is used to treat the different types of appendiceal cancer are highly variable. Chemotherapy treatments appear to show improved efficacy in recent years. Chemotherapy is associated with improved survival for patients with NMAC, MAC and SRCC types of appendiceal cancer. Adequate lymph node sampling results in a survival benefit for appendiceal cancer patients.

## Supplementary Information

**Additional file 1: Table S1.** Risk factors associated with cancer-specific and overall survival for all appendiceal patients diagnosed during 1998–2011. **Table S2.** Risk factors correlated with overall survival in patients with NECs or NETs appendiceal cancer. **Fig. S1.** Effect of chemotherapy on cancer specific and overall survival curves in all patients with appendiceal cancer during different period. (A) Cancer specific survival (1998–2011). (B) Overall survival (1998–2011). (c) Cancer specific survival (2012–2016). (B) Overall survival (2012–2016).

## Data Availability

All data can be drawn from the dataset of the Surveillance, Epidemiology, and End Results (SEER) database (http://www.seer.cancer.gov).
