# Supplementary material for: Chemotherapy in the treatment of different histological types of appendiceal cancers: a SEER based study
Source: BMC Cancer. 2021 Jul 6;21:778. doi: 10.1186/s12885-021-08502-3 (PMC8259079; doi:10.1186/s12885-021-08502-3)

Supplemental data

Table S1. Risk factors associated with cancer-specific and overall survival for all appendiceal patients diagnosed during 1998-2011

| Variable |  | Cancer-specific survival | |  |  | Overall survival | |  |
| --- | --- | --- | --- | --- | --- | --- | --- | --- |
|  | HR (95% CI) | | P value | | HR (95% CI) | | P value | |
| Age |  | |  | |  | |  | |
| ≤60 | 1 | |  | | 1 | |  | |
| >60 | 1.31 (1.12-1.53) | | 0.0006 | | 1.74 (1.54-1.98) | | <.0001 | |
| Gender |  | |  | |  | |  | |
| Male |  | |  | | 1 | |  | |
| Female |  | |  | | 0.81 (0.71-0.92) | | 0.0009 | |
| Marital status |  | |  | |  | |  | |
| Married | 1 | |  | | 1 | |  | |
| Unmarried^#^ | 1.24 (1.05-1.46) | | 0.0122 | | 1.46 (1.28-1.66) | | <.0001 | |
| Unknown | 1.35 (0.91-1.99) | | 0.1312 | | 1.25 (0.9-1.73) | | 0.176 | |
| Region |  | |  | |  | |  | |
| West | 1 | |  | | 1 | |  | |
| South | 1.25 (1.03-1.53) | | 0.0244 | | 1.2 (1.03-1.41) | | 0.0223 | |
| Midwest | 1.33 (1.04-1.71) | | 0.0249 | | 1.21 (0.98-1.48) | | 0.0737 | |
| Northwest | 1.02 (0.82-1.26) | | 0.8696 | | 1.05 (0.88-1.24) | | 0.6045 | |
| Race |  | |  | |  | |  | |
| Black | 1 | |  | | 1 | |  | |
| White | 0.66 (0.52-0.83) | | 0.0005 | | 0.75 (0.62-0.91) | | 0.003 | |
| Other | 0.81 (0.56-1.18) | | 0.2726 | | 0.78 (0.57-1.07) | | 0.1245 | |
| Unknown | 0.53 (0.13-2.19) | | 0.3825 | | 0.53 (0.17-1.68) | | 0.2787 | |
| CEA |  | |  | |  | |  | |
| Negative | 1 | |  | | 1 | |  | |
| Positive | 1.38 (1.09-1.74) | | 0.007 | | 1.41 (1.16-1.71) | | 0.0006 | |
| Unknown | 1.04 (0.85-1.28) | | 0.7079 | | 1.03 (0.87-1.22) | | 0.7338 | |
| Harvested lymph nodes |  | |  | |  | |  | |
| ≤ 12 | 1 | |  | | 1 | |  | |
| >12 | 0.59 (0.5-0.7) | | <.0001 | | 0.59 (0.51-0.67) | | <.0001 | |
| Unknown | 0.76 (0.45-1.29) | | 0.3123 | | 0.66 (0.41-1.06) | | 0.0816 | |
| Histology |  | |  | |  | |  | |
| GCC | 1 | |  | | 1 | |  | |
| MAC | 1.2 (0.83-1.74) | | 0.3328 | | 1.13 (0.86-1.49) | | 0.3928 | |
| MiNENs | 1.61 (1.03-2.52) | | 0.038 | | 1.4 (0.98-1.98) | | 0.0632 | |
| NECs | 0.93 (0.44-1.97) | | 0.8452 | | 1.07 (0.65-1.75) | | 0.7994 | |
| NETs | 0.24 (0.06-1.02) | | 0.0529 | | 0.3 (0.13-0.66) | | 0.003 | |
| NMAC | 2.17 (1.49-3.17) | | <.0001 | | 2.04 (1.53-2.71) | | <.0001 | |
| SRCC | 1.84 (1.25-2.73) | | 0.0022 | | 1.79 (1.32-2.44) | | 0.0002 | |
| T stage |  | |  | |  | |  | |
| T1 | 1 | |  | | 1 | |  | |
| T2 | 0.81 (0.43-1.53) | | 0.5136 | | 0.72 (0.5-1.04) | | 0.0793 | |
| T3 | 1.85 (1.2-2.86) | | 0.0057 | | 1.15 (0.89-1.49) | | 0.2896 | |
| T4 | 2.97 (1.93-4.57) | | <.0001 | | 1.74 (1.34-2.25) | | <.0001 | |
| N stage |  | |  | |  | |  | |
| N0 | 1 | |  | | 1 | |  | |
| N1 | 2.19 (1.78-2.68) | | <.0001 | | 1.86 (1.57-2.21) | | <.0001 | |
| N2 | 2.76 (2.18-3.48) | | <.0001 | | 2.68 (2.2-3.27) | | <.0001 | |
| M stage |  | |  | |  | |  | |
| M0 | 1 | |  | | 1 | |  | |
| M1 | 2.48 (2.04-3.01) | | <.0001 | | 2.05 (1.75-2.4) | | <.0001 | |
| Grade |  | |  | |  | |  | |
| Well differentiated | 1 | |  | | 1 | |  | |
| Moderately differentiated | 1.33 (1.03-1.7) | | 0.0281 | | 1.3 (1.07-1.58) | | 0.008 | |
| Poorly or un-differentiated | 2.68 (2.05-3.51) | | <.0001 | | 2.18 (1.76-2.7) | | <.0001 | |
| Unknown | 1.75 (1.33-2.31) | | <.0001 | | 1.62 (1.31-2) | | <.0001 | |
| Chemotherapy |  | |  | |  | |  | |
| No | 1 | |  | | 1 | |  | |
| Yes | 1.1 (0.92-1.33) | | 0.2937 | | 0.86 (0.74-0.99) | | 0.0385 | |

#Unmarried status including divorced, separated, widowed and unmarried. CI, confidence interval; HR, hazard ratio; SRCC, signet ring cell carcinoma; MAC, mucinous adenocarcinomas; NMAC, non-mucinous adenocarcinoma; MiNENs, mixed neuroendocrine non-neuroendocrine neoplasms; GCC, goblet cell carcinoma; NETs, neuroendocrine tumors; NECs, neuroendocrine carcinomas.

Table S2. Risk factors correlated with overall survival in patients with NECs or NETs appendiceal cancer

| Variable |  | Cancer-specific survival | | | |  | |  | Overall survival | | | |  | |
| --- | --- | --- | --- | --- | --- | --- | --- | --- | --- | --- | --- | --- | --- | --- |
|  | NETs | |  | NECs |  | | NETs | | |  | NECs |  | |  |
|  | HR (95% CI) | | P value | HR (95% CI) | P value | | HR (95% CI) | | | P value | HR (95% CI) | P value | |  |
| Age |  | |  |  |  | |  | | |  |  |  | |  |
| ≤56 |  | |  |  |  | | 1 | | |  | 1 |  | |  |
| >56 |  | |  |  |  | | 1.09 (1.07-1.11) | | | <.0001 | 4.25 (2.09-8.65) | <.0001 | |  |
| Gender |  | |  |  |  | |  | | |  |  |  | |  |
| Male | 1 | |  |  |  | |  | | |  |  |  | |  |
| Female | 0.12 (0.03-0.6) | | 0.0097 |  |  | |  | | |  | 0.4 (0.21-0.76) | 0.0049 | |  |
| Marital status |  | |  |  |  | |  | | |  |  |  | |  |
| Married |  | |  |  |  | | 1 | | |  |  |  | |  |
| Unmarried^#^ |  | |  |  |  | | 1.91 (1.11-3.28) | | | 0.0187 |  |  | |  |
| Unknown |  | |  |  |  | | 0.79 (0.24-2.66) | | | 0.7055 |  |  | |  |
|  |  | |  |  |  | |  | | |  |  |  | |  |
| Race |  | |  |  |  | |  | | |  |  |  | |  |
| Black |  | |  |  |  | |  | | |  | 1 |  | |  |
| White |  | |  |  |  | |  | | |  | 0.37 (0.15-0.92) | 0.032 | |  |
| Other |  | |  |  |  | |  | | |  | 0.81 (0.16-4.18) | 0.7996 | |  |
| Unknown |  | |  |  |  | |  | | |  | 0 | 0.9874 | |  |
| Region |  | |  |  |  | |  | | |  |  |  | |  |
| West |  | |  |  |  | | 1 | | |  |  |  | |  |
| South |  | |  |  |  | | 0.87 (0.42-1.79) | | | 0.6976 |  |  | |  |
| Midwest |  | |  |  |  | | 2.49 (1.19-5.23) | | | 0.0159 |  |  | |  |
| Northwest |  | |  |  |  | | 1.29 (0.64-2.58) | | | 0.4804 |  |  | |  |
| N stage |  | |  |  |  | |  | | |  |  |  | |  |
| N0 |  | |  | 1 |  | |  | | |  | 1 |  | |  |
| N1 |  | |  | 1.91 (0.55-6.63) | 0.3094 | |  | | |  | 0.42 (0.16-1.1) | 0.0775 | |  |
| N2 |  | |  | 6.17 (1.18-32.36) | 0.0313 | |  | | |  | 6.45 (2.12-19.59) | 0.001 | |  |
| Grade |  | |  |  |  | |  | | |  |  |  | |  |
| Well differentiated | 1 | |  | 1 |  | |  | | |  | 1 |  | |  |
| Moderately differentiated | 0 | | 0.992 | 3.28 (0.3-36.31) | 0.3329 | |  | | |  | 1.3 (0.43-3.89) | 0.6397 | |  |
| Poorly or un-differentiated | 22.3 (2.65-187.63) | | 0.0043 | 71.76 (13.94-369.3) | <.0001 | |  | | |  | 3.89 (1.42-10.64) | 0.0081 | |  |
| Unknown | 2.28 (0.54-9.6) | | 0.2607 | 4.15 (0.37-46.21) | 0.247 | |  | | |  | 1.96 (0.65-5.95) | 0.2333 | |  |
| Chemotherapy |  | |  |  |  | |  | | |  |  |  | |  |
| No | 1 | |  | 1 |  | | 1 | | |  | 1 |  | |  |
| Yes | 6.93 (0.85-56.61) | | 0.0707 | 0.88 (0.23-3.33) | 0.8515 | | 2.31 (0.71-7.54) | | | 0.1659 | 0.46 (0.15-1.45) | 0.1844 | |  |

#Unmarried status including divorced, separated, widowed and unmarried. CI, confidence interval; HR, hazard ratio; SRCC, signet ring cell carcinoma; MAC, mucinous adenocarcinomas; NMAC, non-mucinous adenocarcinoma; MiNENs, mixed neuroendocrine non-neuroendocrine neoplasms; GCC, goblet cell carcinoma; NETs, neuroendocrine tumors; NECs, neuroendocrine carcinomas.

Figure S1. Effect of chemotherapy on cancer specific and overall survival curves in all patients with appendiceal cancer during different period. (A) Cancer specific survival (1998-2011). (B) Overall survival (1998-2011). (c) Cancer specific survival (2012-2016). (B) Overall survival (2012-2016).


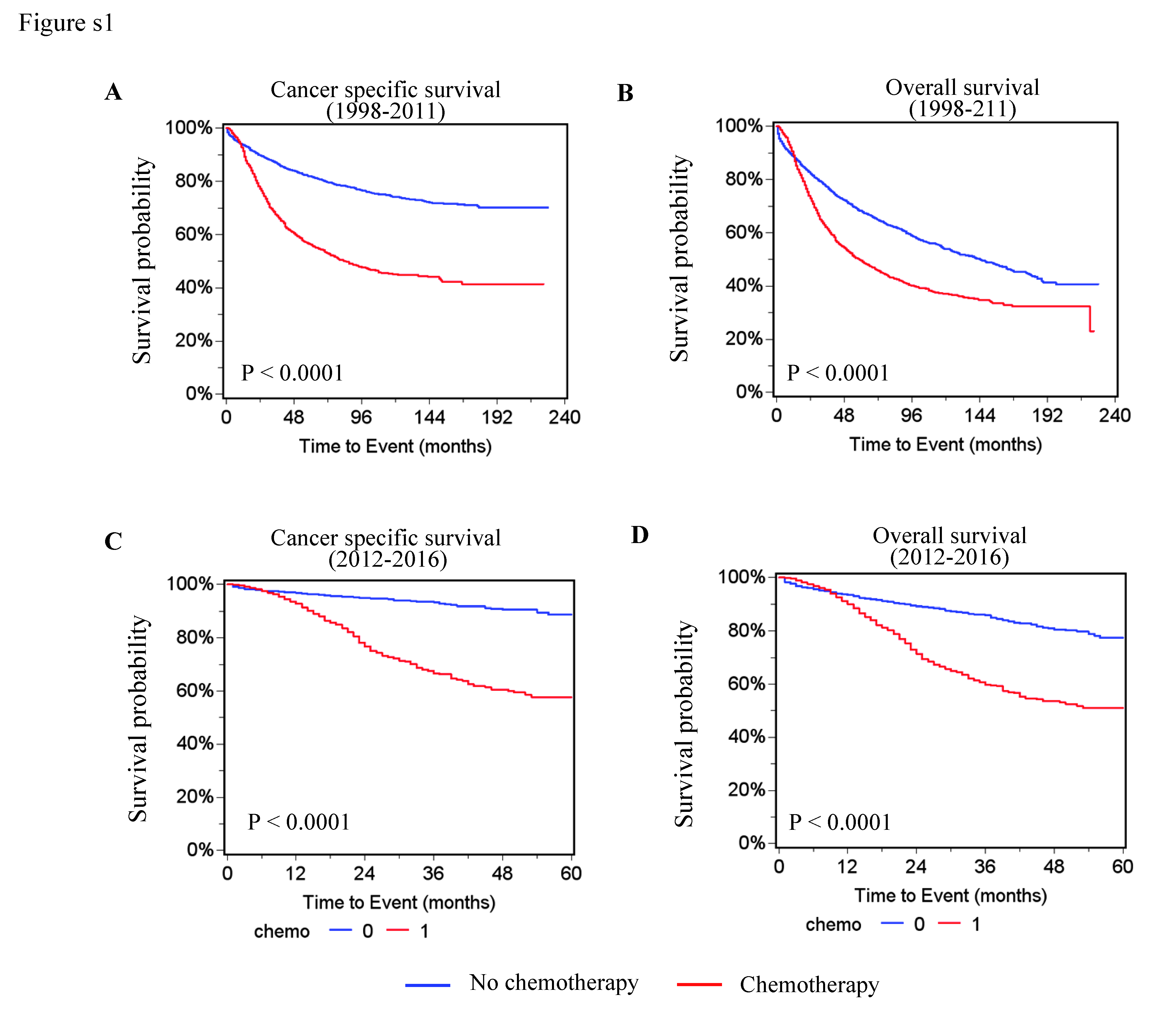

Supplement: Supplementary file 1 — Additional file 1: Table S1. Risk factors associated with cancer-specific and overall survival for all appendiceal patients diagnosed during 1998–2011. Table S2. Risk factors correlated with overall survival in patients with NECs or NETs appendiceal cancer. Fig. S1. Effect of chemotherapy on cancer specific and overall survival curves in all patients with appendiceal cancer during different period. (A) Cancer specific survival (1998–2011). (B) Overall survival (1998–2011). (c) Cancer specific survival (2012–2016). (B) Overall survival (2012–2016). [file 12885_2021_8502_MOESM1_ESM.docx]
